# Low-Dose Strontium-90 Irradiation Is Effective in Preventing the Recurrence of Pterygia: A Ten-Year Study

**DOI:** 10.1371/journal.pone.0043500

**Published:** 2012-08-27

**Authors:** Xue-jiao Qin, Hong-mei Chen, Liang Guo, Yong-yuan Guo

**Affiliations:** Department of Ophthalmology, Qilu Hospital of Shandong University, Jinan, China; National Taiwan University, Taiwan

## Abstract

**Background:**

To study the long-term effects of low-dosage strontium-90 (Sr90) irradiation on the recurrence of pterygium.

**Methodology/Principal Findings:**

One hundred twenty eyes from 104 patients with primary or recurrent pterygia were treated with surgery followed by Sr90 irradiation. In brief, starting on the sixth day after surgery, patients were treated with irradiation three times every other day at a total combined dosage of 2000 cGy to 3000 cGy. Corneal topography was used to evaluate ocular surface regularity before and after treatment. Patient follow-up was performed 2 days, 5 days, 2 weeks, 1 month, 3 months, 1 year, 5 years, and 10 years after surgery. Recurrence of pterygium was not observed in any of the patients in this study. Obvious cataract progression was observed in 6 eyes, which may be due to aging. During follow-up studies, only one eye was reported with dryness and foreign-body sensation. Significant pterygium-induced astigmatism was observed in corneal topography, which decreased after surgery.

**Conclusions/Significance:**

Sr90 irradiation is effective in preventing the recurrence of primary and recurrent pterygia. We recommend delivering a total combined dosage of 2000 cGy to 3000 cGy of Sr90 irradiation administered in three batches every other day starting from the sixth day after surgery. Surgery is important in the rapid recovery of the cornea from pterygium-induced astigmatism.

## Introduction

Pterygium is the abnormal overgrowth of ocular conjunctival tissue. It affects visual acuity when it spreads to the cornea, especially when near the level of the pupil margin. Although the pathogenesis of pterygium is not clearly understood, ultraviolet (UV) light is widely accepted as its single most important etiological factor. UV radiation may trigger a chain of events that can damage DNA, RNA, and the extracellular matrix [1]. The incidence of pterygia is high in tropical regions, especially in the equatorial zone between latitudes of 30 degrees north and south [2]. Pterygium treatments are complicated because of the high rate of recurrence of the disease [3]. Mitomycin C, thiotepa, amniotic membrane transplantation, limbal conjunctival auto graft transplantation, and β irradiation have been used in the management of pterygium recurrence after resection [4], [5], [6], [7]. Bevacizumab, a novel monoclonal antibody against vascular endothelial growth factor, has also been used to prevent recurrence [8]. β-irradiation, such as that using strontium-90 (Sr90), has been used for half a century for the prevention of pterygium recurrence. However, although the therapeutic effects of irradiation are obvious, side effects, such as scleral necrosis, cataracts, and ocular infection, that may eventually cause blindness have also been noted [9]–[15].

Most irradiation treatments have been typically carried out immediately after surgery and with a single dose of about 2500 cGy. In our study, we used Sr90 irradiation to prevent pterygium recurrence through a safer and relatively more convenient strategy, that is, at a total dose of 2000 cGy to 3000 cGy delivered in three batches starting from the sixth day after surgery. A 10-year follow-up was carried out, and the results obtained are encouraging.

## Methods

Ethical statement: The study complies with the protocols reviewed and approved by the independent ethics committee of the Qilu Hospital of Shandong University and the tenets of the Declaration of Helsinki. The clinical trial registration number of this study is ChiCTR-ONC-12001981. Signed informed consent was obtained from all participants.

Patients with pterygium were recruited from December 1999 to December 2000 at the Qilu Hospital of Shandong University. The inclusion criteria were as follows: (1) Ability to visit the Shandong Academy of Medical Sciences and finish the three irradiation treatments; (2) willingness to be followed up; and (3) no serious systemic diseases, especially connective tissue diseases, such as systemic lupus erythematosus, Sjogren’s syndrome or congenital collagen defects . One hundred twenty eyes from 104 patients were studied. All of the patients enrolled were Chinese aged from 23 years to 78 years, with a mean age of 55.19 years ±8.22 years. Cataracts were observed in 52 eyes (43.3%) with different severities at the basal level. Sixteen eyes presented with recurrent pterygia, one of which had relapsed twice and had undergone a third surgery before being subjected to Sr90 irradiation. All but one patient received surgery, and all patients received Sr90 irradiation following surgery, if any. The surgery was a combination of two classical procedures : Excision [16] + transposition [17], briefly , it involved a pterygium resection from the corneal surface, removal of the head and neck parts of the pterygium, and translocation of the remaining body part downward and its embedment under the conjunctiva. All surgeries were conducted by the same ophthalmologist. Tobramycindexamethasone eye drops were applied three times a day after surgery for 2 weeks.

Sr90 irradiation was carried out at the Shandong Academy of Medical Sciences using a Sr90 ophthalmic bowl-shaped applicator (Amersham, Germany). The outer diameter of the active area was 12 mm and the sensitive area of the probe was 0.5 mm thick and approximately 3 mm in diameter. A mimic picture of the applicator was shown in the supporting file Figure S1. Irradiation was conducted starting on the sixth day after the surgery, when the sutures of the conjunctiva were removed. Irradiation was carried out in three batches every other day, each at a dosage of 700 cGy (A timer was set for application duration according to the dose rate of the source at the treating time, the maximum error of dose rate was ±10%), if the arc length of the pterygium at the corneal margin was over 3mm, two irradiations were applied side by side at the first treatment. So a total combined dosage ranging from 2000 cGy to 3000 cGy was applied.

Patients were followed up 2 days, 5 days (at which time the irradiation was conducted), 2 weeks, 1 month, 3 months, 1 year, 5 years, and 10 years after surgery.

Corneal topographic examinations were conducted in 18 eyes from 17 patients (EyeSys System 2000 Corneal Analysis System, EyeSys, USA) before and 1 week after surgery.

Statistical analysis was performed using SPSS 16 (SPSS, Inc.). Comparisons between parametric changes in corneal topography before and after surgery was done using paired samples t-test with p<0.05 signifying statistical significance.

## Results

**Figure 1:**
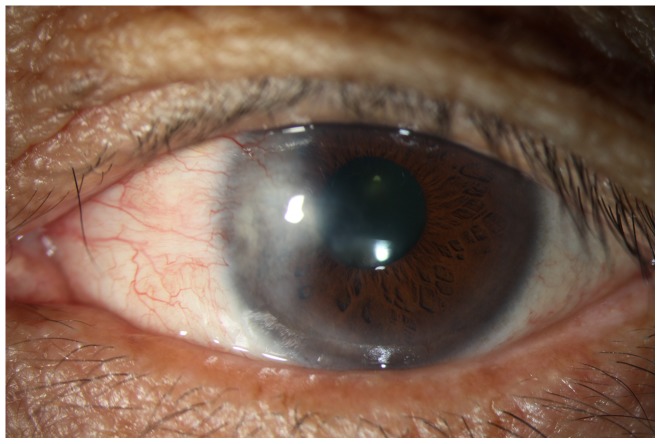
Ocular surface of a patient who underwent pterygium surgery three times before receiving Sr90 irradiation. A visual acuity of 18/20 was observed at the 10-year follow-up.

All of the eyes showed conjunctival congestion at the 2-day and 5-day follow-up. Six eyes (5%) showed delayed corneal epithelial healing 2 weeks post surgery, which were resolved at 1 month post-surgery follow-up or earlier. The number of eyes and the major ocular conditions are shown in Table 1. Five patients died before the 10-year follow-up. At the 10-year follow-up, 4 eyes (3.48%) from 4 patients aged 65 years or older had undergone cataract surgery. Two eyes (1.74%) developed obviously progressed cataracts. We did not observe pterygium recurrence in any of the patients in the study, including the one without surgery. Figure 1 shows the ocular surface of the patient who underwent surgery three times before receiving Sr90 irradiation.

**Table 1:** Number of eyes (%) with different ocular manifestations in the follow-ups.

Ocular manifestations					
Follow-up	Conjunctival congestion	Corneal epithelial defects	Ocular infection	Ocular ulcer/necrosis	Cataract development
2 days	120 (100%)	32 (26.7%)	0	0	0
5 days	82 (68.3%)	0	0	0	0
2 weeks	12 (10%)	6 (5%)	0	0	0
1 month	5 (4.2%)	0	0	0	0
3 months	0	0	0	0	0
1 year	0	0	0	0	0
5 years	0	0	0	0	3 (2.5%)
10 years	0	0	0	0	6 (5.2%)

Corneal topographic examinations showed significant preoperative with-the-role astigmatism and a statistically significant reduction in astigmatism after surgery. Data are shown in Table 2 (paired samples T-test).

**Table 2:** Changes in corneal topographic refraction before and after pterygial operation.

	Before	After	p-value*
Steepest radium(D)	44.42±1.59	44.98±1.73	*0.0407*
Flattest radium(D)	42.51±1.48	43.93±1.50	***0.0007***
Mean refraction(D)	43.60±1.97	44.14±1.74	0.1158
Central refraction(D)	43.90±1.83	44.35±1.75	0.0559
Astigmatism (D)	1.91±1.10	1.05±0.91	***0.0034***

* paired samples t-test.

## Discussion

Sr90 only emits β-irradiation. It decreases cell division, prolongs the inter-division period, blocks the microvessels, and thus induces rapid cell decay. Tissue permeability of Sr90 is low, the percentage depth dose in tissue drops to 41% at 1 mm (the conjunctiva level), 9% at 3 mm (the sclera level) and almost zero at 5 mm (The Lens level) [18]. Beta- irradiation of pterygium is a traditional treatment with many new methods of delivery [19]. The time and amount of Sr90 application are the foci of radiation management. Traditionally, Sr90 irradiation is applied immediately after surgery with a single dose ranging from 750 cGy to 5200 cGy. The most serious side effects of single-dose irradiation include scleral ulceration or even perforation. Cataract development has also been noted in long-term follow-up [13], [20]. The proposed method of applying irradiation in fractions thus appears to be a safer approach [14], [21], [22]. The optimal time for postoperative irradiation is also controversial. Aswad [23] found immediate postoperative irradiation was associated with a lower rate of recurrence than irradiation given 4 days postoperatively. Bernstein and Unger [24] obtained the best results when irradiation was given before the fourth postoperative day. Isohashi et al. [9] suggested that performing the irradiation at a certain interval after surgery might improve therapy outcome. Cooper and Lerch [25] found that radiation therapy begun 4 days after surgery resulted in fewer recurrences than radiation administered within 3 days of surgery. In our study, we assigned the irradiation 6 days after surgery, when the conjunctival lesion caused by surgery had healed, and the irradiative effects were favourable. Even for the case without surgery, Sr90 irradiation alone could effectively control the pterygium. Pajic and Greiner treated 54 primary pterygia with an exclusive Sr90/yttrium-90 beta irradiation up to a total dose of 50 Gy. In the long term follow-up (averaging 112+/-88 months and ranging from 12 months to 321 months), a reduction in the size of every pterygium was observed, and none of the 54 pterygia developed recurrent growth. In addition, no patient manifested any late side effects [26]. These studies suggest that the interval before irradiation may not be crucial and a slight delay in application may have the advantage of thorough wound healing and less chances of infection. We used a moderate dosage of 2000 cGy to 3000 cGy delivered three times in a staggered system, the larger dose was assigned to lesions over 3mm. This was similar to a semi-personalised treatment, because the severity of the disease differed among patients, theoretically applying the same dose to extremely different cases cannot produce the same result. At the 10-year follow-up, this treatment regimen proved safer than and as effective as single-dose irradiation, consistent with the regimen proposed by Ali et al. [27], who reviewed more than 6000 cases of pterygia treated with different fractionation schemes and concluded that 30 Gy in 3 fractions over 2 weeks to 3 weeks is effective and safe. No recurrence was observed in any of our patients, including the one who received surgery three times. For the lone patient without surgery, the pterygium decreased by the 10-year follow-up visit. The recurrence rate obtained is lower than the commonly reported 10%. This result may be independent of the extent of pterygia. The sizes of the pterygia cases in our study (with a mean horizontal length of 3.68 mm ±1.66 mm) were larger than those reported by Pavilack et al. (with a mean horizontal length of 2.00 mm ±1.00 mm) [28]. The “triad” procedure (i.e., Excision + transposition + irradiation) and avoidance of repeated scrubbing during operation may have also contributed to the reduction in recurrence. In our clinical experience, for patients without irradiation, the recurrence rate for primary pterygia was about 10% but not more than 20% (not a statistical result). The inclusion criteria in this study were mainly focused on the patients’ availability. Whether there was a low risk group–which could be treated exclusively with surgery, or a high risk group–which should be treated with a combination of surgery and brachytherapy is to be further investigated. Six patients developed evident cataracts. We believe that this finding cannot be completely attributed to irradiation, considering that the patients were all at an advanced age and the cataract incidence was low. Recently, Viani et al. [29] reported the result of treatment with two different fractionation schedules of β-irradiation, the high fractionation group was assigned 5Gy within 7 fractions (7d/wk, total dose 35Gy), the low fractionation group was assigned 2Gy within 10 fractions (5d/wk, total dose 20Gy), they found no significant difference in the control rate (the 3-year recurrence rate in both group was less than 8%), but the low-dose fractionation group had better cosmetic effects and fewer symptoms. They also calculated the biological effective dose (BED): BED = *nd* [1+ *d*/(*α/β*)] [30], and got a very low BED of 24 Gy_10_ for the low fractionation group. But the minimal radiation dose which can control pterygia is still to be confined. During the entire follow-up period, the only adverse effect was observed in one woman who reported to have dry eyes and foreign body sensation but had an otherwise intact corneal epithelium. The good control rate and minimal side-effects proved our treatment regimen could be advocated. Corneal topographic examinations showed that astigmatism caused by pterygium decreased rapidly after surgery.

This study proves that Sr90 irradiation is effective against the recurrence of primary and recurrent pterygia. A total combined dose of 2000 cGy to 3000 cGy delivered in three fractions every other day starting on the sixth day post-surgery is recommended. As well, a “triad” treatment (i.e., excision, transposition, and irradiation) is recommended for the management of pterygium.

## Supporting Information

Figure S1 **Mimic picture of the applicator.**
(TIF)
