# Supplementary figures and images for: Low-Dose Strontium-90 Irradiation Is Effective in Preventing the Recurrence of Pterygia: A Ten-Year Study
Source: PLoS One. 2012 Aug 27;7(8):e43500. doi: 10.1371/journal.pone.0043500 (PMC3428357; doi:10.1371/journal.pone.0043500)

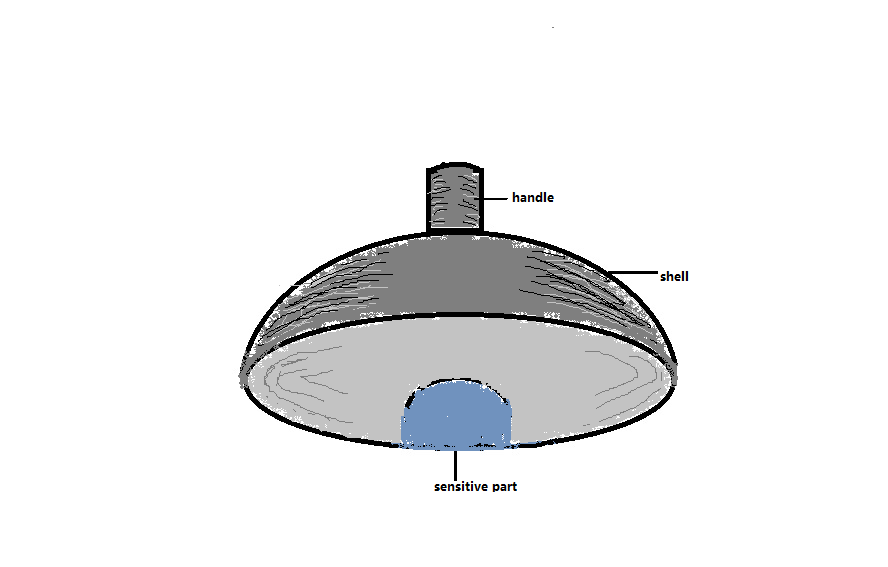

Supplement: Figure S1 — Mimic picture of the applicator. (TIF) [file pone.0043500.s001.tif]
